# Amerindian genetic ancestry as a risk factor for tuberculosis in an amazonian population

**DOI:** 10.1371/journal.pone.0236033

**Published:** 2020-07-16

**Authors:** Diana Feio da Veiga Borges Leal, Mayara Natália Santana da Silva, Débora Cristina Ricardo de Oliveira Fernandes, Juliana Carla Gomes Rodrigues, Maria Clara da Costa Barros, Pablo Diego do Carmo Pinto, Lucas Favacho Pastana, Cleonardo Augusto da Silva, Marianne Rodrigues Fernandes, Paulo Pimentel de Assumpção, Sidney Emanuel Batista dos Santos, Ney Pereira Carneiro dos Santos

**Affiliations:** 1 Núcleo de Pesquisas em Oncologia, Universidade Federal do Pará, Belém, Pará, Brazil; 2 Laboratório de Genética Humana e Médica, Universidade Federal do Pará, Belém, Pará, Brazil; Jamia Hamdard, INDIA

## Abstract

In recent years, the incidence of tuberculosis (TB) has declined worldwide, although this disease still occurs at relatively high rates in Amerindian populations. This suggests that the genetic ancestry of Amerindians may be an important factor in the development of infections, and may account for at least some of the variation in infection rates in the different populations. The present study investigated the potential influence of Amerindian genetic ancestry on susceptibility to tuberculosis in an Amazon population. The study included 280 patients diagnosed with tuberculosis and 138 asymptomatic hospital employees with no history of TB, but who were in contact with bacterially active TB patients. Ancestry analysis was run on a set of 61 Ancestry-Informative Markers to estimate European, African, and Amerindian genetic ancestry using STRUCTURE v2.2. The TB group had significantly higher Amerindian ancestry in comparison with the control group, and significantly lower European ancestry. Amerindian ancestry in the 20–60% range was found to be the principal risk factor for increased susceptibility to TB. The results of the study indicate that Amerindian ancestry is an important risk factor for susceptibility to TB in the admixed population of the Brazilian Amazon region.

## Introduction

Tuberculosis (TB) is an infectious disease caused by the *Mycobacterium tuberculosis* bacillus (MTB). In 2018, 10 million incident cases of TB were registered, resulting in 1.2 million TB deaths, a number higher than that caused by Human Immunodeficiency Virus [1,2]. While the incidence of TB and the total number of deaths, worldwide, have declined slightly in recent years, TB continues to be a major endemic concern for the amerindian people of the Brazilian Amazon [3,4].

In amerindian population tend to have much higher rates of TB in comparison with the general (non-indigenous) population [5–7]. This disparity has been attributed to multiple factors, including environmental and socioeconomic variables, co-infections with other pathogens, and the genetics of the host [8]. The profile of genomic ancestry is known to be a determining factor in the susceptibility of some groups to certain diseases, as well as their response to treatment [9].

Brazil is one of the most heterogeneous countries in the world with a population formed primarily by the admixture of three continental groups: European (EUR), African (AFR), and Amerindian, AMR [10]. Overall, EUR ancestry predominates in Brazil (62.4%), while AFR ancestry is the second most prevalent (22.6%), and AMR, the least common, at 14.7% [11]. In northern Brazil, however, the two minor ancestries are reversed, with AMR increasing to 28%, while EUR decreases to 60% and AFR to 12% [12–15].

Genetic studies have indicated that populations of different ancestry, in particular AMR, may be relatively more susceptible to certain diseases than other populations [15–18]. In Mexican and Peruvian populations, for example, AMR ancestry was associated with increased risk for systemic lupus erythematosus [16]. In northern Brazil, AMR ancestry has been associated with susceptibility to the development of leprosy, including the severe clinical form [17], as well as an increased prevalence of hepatitis B and C [18], and an increased risk of developing acute lymphoblastic leukemia [15].

While these studies have focused on different diseases and populations from northern Brazil and other regions of Latin America, no data are available on the incidence of TB or its association with genetic factors in the population of the Amazon region. Given this, the present study investigated the potential role of AMR genetic ancestry in the susceptibility to TB infection of a highly admixed Amazonian population.

## Material and methods

### Study populations

This is a case-control study was conducted between March 2008 and March 2010 at João de Barros Barreto Hospital, in Belém city, Brazil. Two hundred eighty TB patients were enrolled in case group and one hundred and thirty-eight in control group. The case group was composed of participants aged over 18 years with confirmed pulmonary TB according to the World Health Organization (WHO). Exclusion criteria was individuals with confirmed extra-pulmonary TB. The control group was composed of participants who were employees from the Hospital in contact with active TB patients for at least five years with neither symptoms nor diagnostic confirmed of TB.

### Ethical aspects

The protocol adopted in the present study was approved by the Committee for Research Ethics of the Federal University of Pará (approval no. 350507). Informed consent was obtained from all the participants of the study.

### Extraction and quantification of the DNA

The genomic DNA was isolated from peripheral blood leukocytes using the Biopur mini spin plus kit (Biometrix Inc., San Francisco, CA, USA) and quantified with a Thermo Scientific NanoDrop 1000 spectrophotometer (NanoDrop Technologies, Wilmington, DE).

### Ancestry analysis

A set of 61 Ancestry-Informative Markers (AIMs) was used to estimate the proportion of three different ancestries: European (EUR), African (AFR), and Amerindian (AMR). These indel-type markers were previously described [12,19]. The AIMs were genotyped by Multiplex PCR, followed by capillary electrophoresis with fragment analysis. The multiplex amplifications were run using the QIAGEN Multiplex PCR kit (QIAGEN, Germany), with the PCR being run in an ABI Veriti thermocycler (Life Technologies, USA), followed by the capillary electrophoresis protocol. The DNA fragments were separated using an ABI PRISM 3130 Genetic Analyzer and peak reads were obtained in GeneMapper ID v3.2 software (Life Technologies). The experiment was replicated twice.

### Statistical analysis

The samples were analyzed in STRUCTURE v2.2 to determine the relative contribution of EUR, AFR and AMR ancestries to the genetic profile of each individual [20,21]. Additional statistical analyses were made using the software RStudio v.3.6.3 with the basic statistics package (*stats*) for the R language. The analysis of a categorical non-parametric clinical data (sex) was performed with the Fisher test, while the analysis of an ordinal non-parametric clinical data (age) was performed with Mann-Whitney test. Ancestry informative markers (AIM) was analyzed using the multivariate logistic regression model. Clinical variables with significant results were used as covariates. All tests were two-tailed and *p-value* ≤ 0.05 was considered statistically significant.

## Results

Two hundred and eighty tuberculosis patients and one hundred thirty-eight control individuals were analyzed. Table 1 presents the demographic and clinical characteristics of the case and control group. The results showed significant differences when the cases were compared against controls in relation to sex and African, European and Amerindian ancestry. The female gender was predominant in the case and control group with 52% and 85% respectively (*p* = 1.33x10^-11^).

**Table 1:** Demographic and clinical characteristics of the case and control groups selected for the present study.

VARIABLE	TUBERCULOSIS (n = 280)	CONTROL (n = 138)	*p-value*
Age (years)	47 ± 21.1	52 ± 14.4	0.052 ^a^
Sex (male/female)	134 (48%) / 146 (52%)	21 (15%) / 117 (85%)	1.33x10^-11^ ^b^
AFR ancestry	0.254 ± 0.12	0.216 ± 0.11	0.004 ^c^
EUR ancestry	0.409 ± 0.13	0.499 ± 0.17	7.56x10^-7^ ^c^
AMR ancestry	0.337 ± 0.13	0.285 ± 0.14	0.001 ^c^

^a^Mann-Whitney test

^b^Fisher's exact test

^c^Multivariate regression logistic with sex as a covariate. The data are presented as the median ± standard deviation.

The ethnic composition of the case group was 25.4%% African, 40.9% European, and 33.7% Amerindian, while in the control group composition was 21.6% African, 49.9% European, 28.5% Amerindian (Table 1). These results suggested an increased contribution from Amerindian ancestry (*p* = 0.001) and a loss of contribution from European ancestry (*p* <0.001) in the case group compared to the control group.

**Fig 1:**
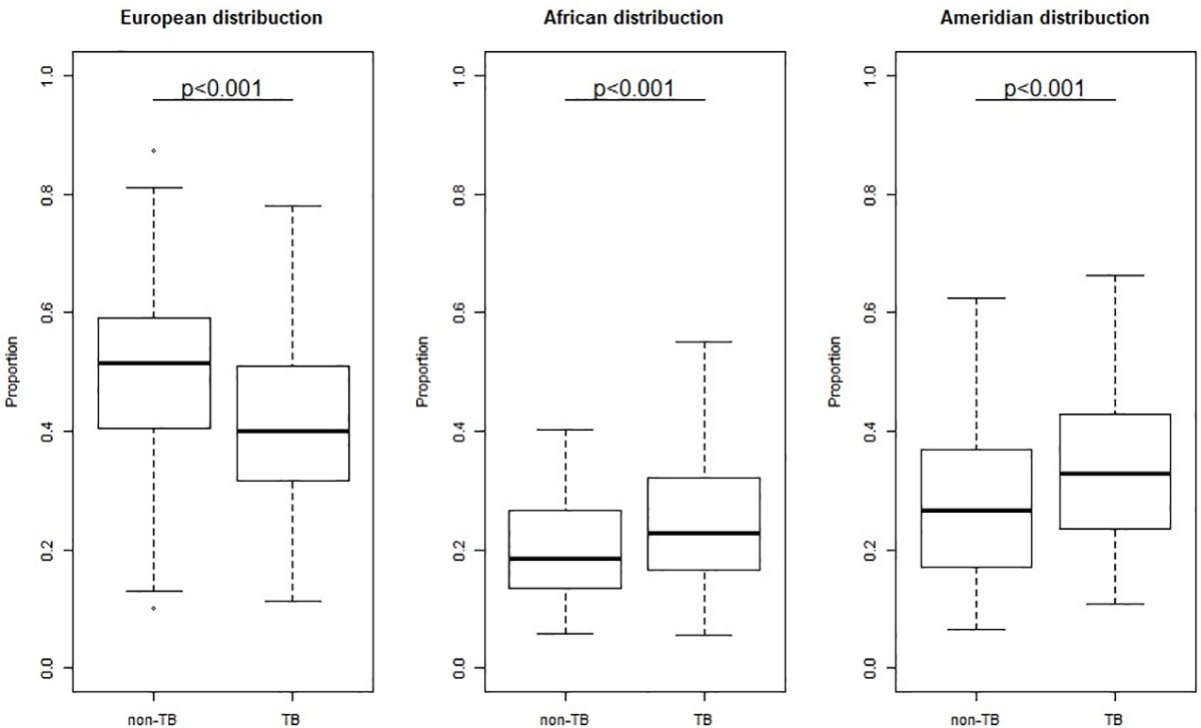
Distribution of the three principal ancestries (EUR, AFR, and AMR) in the case (TB) and control (non-TB) groups analyzed in the present study. Significance determined by multivariate regression logistic with sex as a covariate.

Distribution of the three principal ancestries, EUR, AFR, and AMR, in the case and control groups was presented in Fig 1. The ancestry of the case-control groups was investigated by comparing interdependent components. These results indicated that EUR ancestry was less amply distributed in the TB group, whereas the AMR and AFR ancestries of this group were higher than those of the control group (Fig 1).

**Fig 2:**
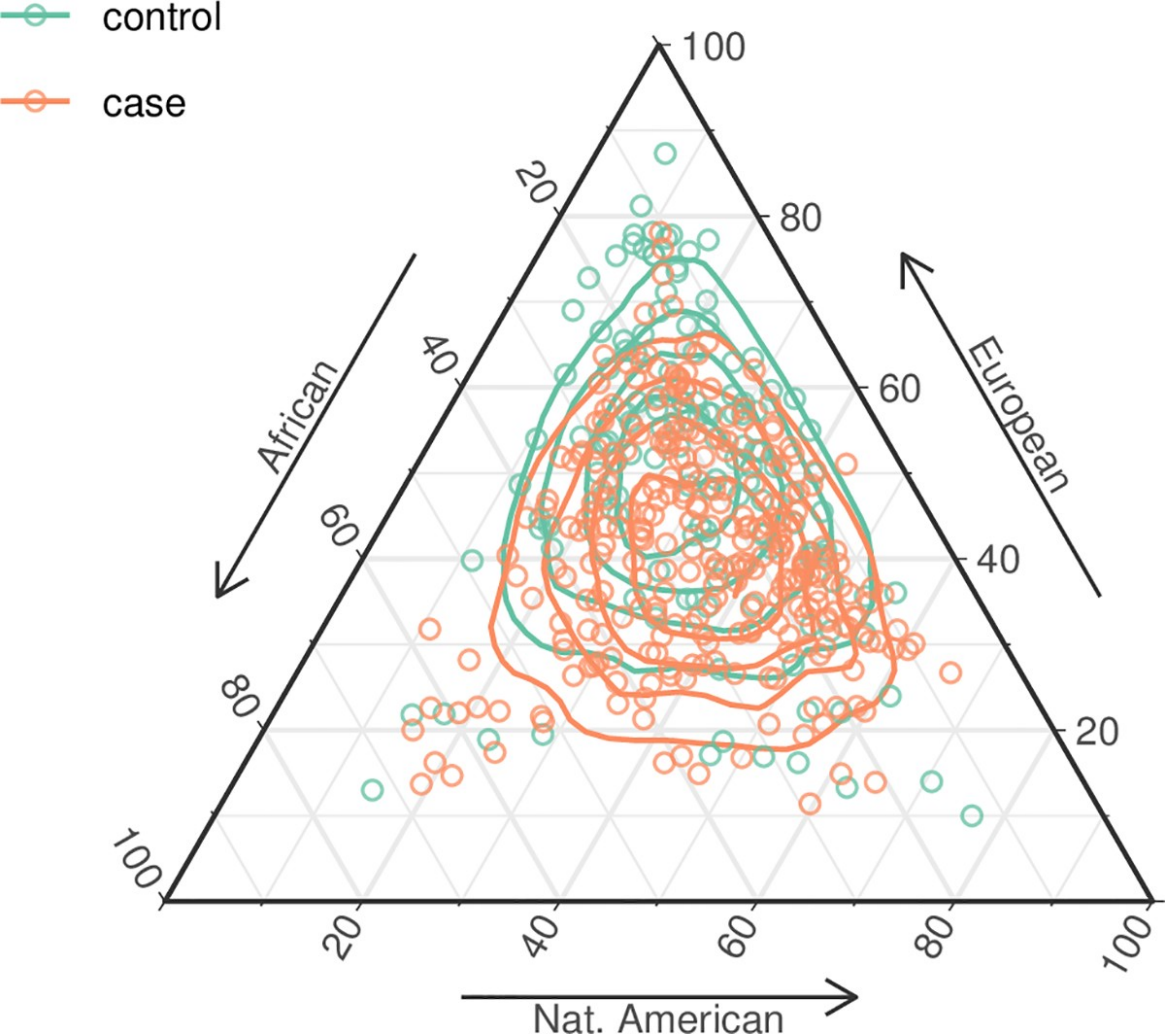
Estimates of genetic admixture in the case and control individuals analyzed in the present study.

The individual interethnic profiles of the study participants were presented in Fig 2. In this plot, the TB patients were represented by orange dots and the control subjects by green dots, and their respective positions within the vertices of the triangle correspond to the relative contribution of EUR, AFR and AMR ancestry. This analysis provided a visual representation of the different ancestries of the subjects of the two groups. Most of the TB patients were closer to the lower part of the triangle, which reflects their greater average AMR ancestry. By contrast, most individuals in the control group were closer to the upper portion of the triangle, reflecting their greater EUR ancestry.

**Fig 3:**
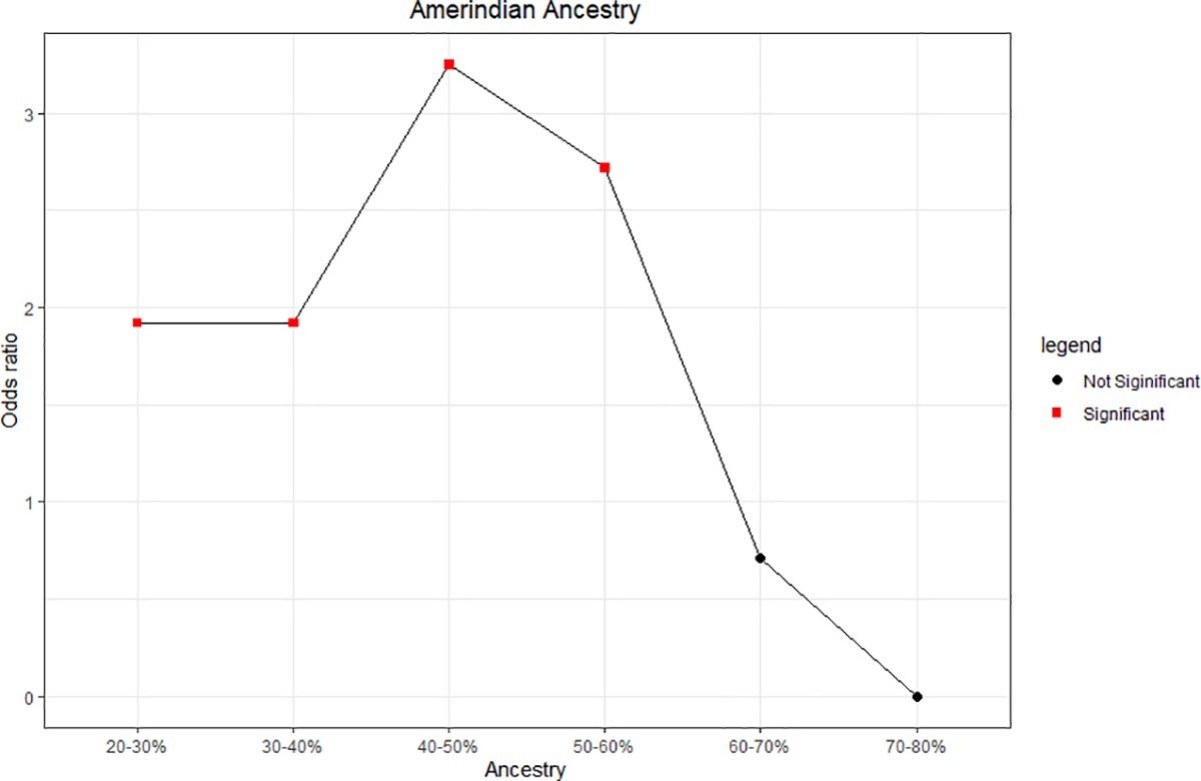
Variation in the odds ratios recorded for different percentages of Amerindian ancestry as a risk factor for tuberculosis. Significance as determined by multivariate regression logistic with sex as a covariate.

The influence of the range of AMR ancestry on the susceptibility of individuals to TB was assessed using multivariate regression logistic model with sex as a covariate (Fig 3). This analysis indicated that AMR ancestry in the 20% to 60% range was a risk factor for susceptibility to TB, with the risk peaking in the upper portion of this range (40–50%). When the contribution of AMR ancestry was in the 20–30% range, the estimated risk effect was 1.9 (OR = 1.92, 95%CI = 1.03–3.63; *p* = 0.041), in the 30–40% was 1.92 (OR = 1.91, 95% CI = 1.01–3.66, *p* = 0.047). Risk peaked in the 40–50% range, where the Odds Ratio for TB infection was 3.25 (95%CI = 1.56–7.01, *p* = 0.002) and decreased in the 50–60% range where the OR was 2.72 (95% CI = 1.09–7.30, *p* = 0.037). The lack of any significant risk effect at the extremes of this range (below 20% AMR ancestry and above 60%) was probably due to the restrictively small sample sizes for these levels of ancestry, which limited the discriminative power of the statistical tests.

## Discussion

In the present study, we investigated the potential role of Amerindian genomic ancestry in the susceptibility to tuberculosis of individuals from the Brazilian Amazon region. This population is admixed from three groups, of European (EUR), African (AFR), and Amerindian (AMR) ancestry. Brazil’s interethnic mix is ​​one of the most heterogeneous anywhere in the world, resulting in a uniquely diverse population [22]. The population of Brazil is ethnically distinct from the populations of both neighboring countries and other continents [23].

Tuberculosis still occurs at high rates and is one of the leading causes of disease-related mortality, worldwide [24]. The northern (Amazon) region of Brazil has relatively high rates of TB in comparison with the other regions of the country [25]. That is probably because of factors such as human polygenic variability which is an important determinant of the outcome of infection with MTB [26]. Epidemiological studies have evaluated high incidence of TB disease in Amerindian [27,28] and North of Brazil has high contribution of AMR ancestry [13] so these factors may be associated and consequently have important clinical implications.

The present study evaluated the role of genetic ancestry in the susceptibility to TB in a highly miscegenated population. The results of the genetic analysis of the three ancestral groups, i.e., European (EUR), African (AFR), and Amerindian (AMR), found significant differences among these groups. In particular, the AMR ancestry of the TB group was significantly higher than that of the control group and increasing AMR ancestry also contributed to an increased predisposition for infection by TB.

The highly heterogenous population of Brazil may be relatively more susceptible to some diseases than other populations, and the results of the present study further reinforce the need for the identification of the EUR, AFR and AMR ancestries in a given population in order to best predict the health risks of each individual [29]. Norris *et al*. [30] reported significant variation in the pathways of immunity, metabolism, and disease in distinct populations. These factors may contribute to the health-related disparities found in substructured Latin American populations, in particular, that different ancestral profiles may have distinct predispositions to specific diseases.

Chimusa *et al*. [31] reported an association of ancestry with TB susceptibility in an African population and concluded that this ancestry conferred a high risk of contracting the disease. Daya *et al*. [32] also concluded that increased AFR ancestry and decreased EUR ancestry were associated with an increased risk of developing TB.

A number of studies in Brazil have also highlighted the importance of the role of ancestry in the susceptibility of a population to disease, including the study of Pinto *et al*. [17], which identified a set of potential genetic markers related to susceptibility to leprosy, influenced in particular by AFR and EUR genomic ancestry. Carvalho *et al*. [33] also found an association between AMR ancestry and an increased risk of developing acute lymphoblastic leukemia in the population of Brazilian Amazonia. Lopes *et al*. [34] also concluded that reduced EUR genetic ancestry and increased AFR ancestry had a significant effect on susceptibility to chronic periodontitis.

Amerindian populations are immunologically distinct from non-Amerindian ones, with reduced variability in some immune system genes such as *HLA*, *KM*, *GM*, and *KIR* [35–39]. This implies that Amerindian populations may have a distinct immune response from the general population, in particular, that the Th2-type immune response is predominant in this group [27]. The Th2-type response induces a humoral response, which is inefficient in the defense of the organism against intracellular pathogens, such as MTB [40,41].

The results of the present study show that Amerindian genomic ancestry is a risk factor for tuberculosis, although we believe that this influence does not arise from the neutral polymorphisms used to infer genomic ancestry, but rather, reflects the low genetic diversity of the immune response in Amerindian populations and, in turn, of the admixed population with a high AMR ancestry. This means that individuals who have a higher Amerindian genomic ancestry have a higher risk of developing tuberculosis.

The incidence of TB in Amerindian populations has been amply studied from both epidemiological and socioeconomic viewpoints [5,28,42,43], albeit with a deficit in genetic research. Given this, the present study was a pioneering investigation of the association of Amerindian genetic ancestry with TB in the population of Brazilian Amazonia, which highlighted the importance of disease-oriented genomic ancestry studies in admixed populations. Our findings indicate that Amerindian ancestry is an important risk factor for TB in the admixed populations of the Brazilian Amazon region. Further research into the relationship between genetic ancestry and the prevalence of infectious diseases may provide important practical insights for the diagnosis, prevention, and treatment of diseases in different populations.

There were some limitations in the study. First, the sample sizes were relatively small and the case and control groups were individuals with high admixture of ancestry and genetic background is different from other populations, so findings might not generalizable to the population of Brazil. Second, the study was unable to obtain sufficient data related to socio-economic factor, cigarette smoking, alcohol consumption, in order to perform a stratified analysis. Despite these drawbacks, it could detect association between genetic ancestry and tuberculosis. There is lack in the literature about the influence of Amerindian ancestry in the risk or protection on infectious disease as tuberculosis. In future work, there is an interest in whole exome-sequencing in admixture population with high proportion of Amerindian ancestry to investigate immune gene variants associated to susceptibility to pulmonary tuberculosis infection.

## Supporting information

S1 Table Distribution table of patients according to the Amerindian ancestry range, with p-value, odds ratio and 95% confidence interval. All statistical data were performed using the multivariate logistic regression model with the sex control variable.
(DOCX)

S2 Table Tabela de Dados Brutos. (PDF)

## References

[1] Rashak HA, Sánchez-Pérez HJ, Abdelbary BE, Bencomo-Alerm A, Enriquez-Ríos N, Gómez-Velasco A, et al Diabetes, undernutrition, migration and indigenous communities: tuberculosis in Chiapas, Mexico. Epidemiol Infect. 2019;147: e71 10.1017/S0950268818003461 30869023 PMC6518577

[2] WHO. Tuberculosis. 2019 [cited 11 Apr 2019]. Available: https://www.who.int/en/news-room/fact-sheets/detail/tuberculosis

[3] Basta PC, Coimbra CEA, Escobar AL, Santos RV, Alves LCC, Fonseca L de S. Survey for tuberculosis in an indigenous population of Amazonia: the Suruí of Rondônia, Brazil. Trans R Soc Trop Med Hyg. 2006;100: 579–585. 10.1016/j.trstmh.2005.07.014 16274716

[4] Basta PC, Coimbra Jr CEA, Welch JR, Corrêa Alves LC, Santos RV, Bastos Camacho LA. Tuberculosis among the Xavante Indians of the Brazilian Amazon: An epidemiological and ethnographic assessment. Annals of Human Biology. 2010;37: 643–657. 10.3109/03014460903524451 20113213

[5] Malacarne J, Kolte IV, Freitas LP, Orellana JDY, Souza MLP de, Souza-Santos R, et al Factors associated with TB in an indigenous population in Brazil: the effect of a cash transfer program. Rev Inst Med Trop Sao Paulo. 2018;60: e63 10.1590/S1678-9946201860063 30379230 PMC6201742

[6] Narasimhan P, Wood J, MacIntyre CR, Mathai D. Risk Factors for Tuberculosis. Pulm Med. 2013;2013 10.1155/2013/828939 23476764 PMC3583136

[7] Tollefson D, Bloss E, Fanning A, Redd JT, Barker K, McCray E. Burden of tuberculosis in indigenous peoples globally: a systematic review. Int J Tuberc Lung Dis. 2013;17: 1139–1150. 10.5588/ijtld.12.0385 23823137 PMC6057791

[8] Viana PV de S, Gonçalves MJF, Basta PC. Ethnic and Racial Inequalities in Notified Cases of Tuberculosis in Brazil. PLOS ONE. 2016;11: e0154658 10.1371/journal.pone.0154658 27176911 PMC4866698

[9] Zhang W, Dolan ME. Ancestry-related differences in gene expression: findings may enhance understanding of health disparities between populations. Pharmacogenomics. 2008;9: 489–492. 10.2217/14622416.9.5.489 18466094 PMC2665165

[10] Andrade RB, Amador MAT, Cavalcante GC, Leitão LPC, Fernandes MR, Modesto AAC, et al Estimating Asian Contribution to the Brazilian Population: A New Application of a Validated Set of 61 Ancestry Informative Markers. G3 (Bethesda). 2018;8: 3577–3582. 10.1534/g3.118.200650 30185426 PMC6222592

[11] de Souza AM, Resende SS, de Sousa TN, de Brito CFA. A systematic scoping review of the genetic ancestry of the Brazilian population. Genet Mol Biol. 2019;42: 495–508. 10.1590/1678-4685-GMB-2018-0076 31188926 PMC6905439

[12] Santos NPC, Ribeiro‐Rodrigues EM, Ribeiro‐dos‐Santos ÂKC, Pereira R, Gusmão L, Amorim A, et al Assessing individual interethnic admixture and population substructure using a 48–insertion-deletion (INSEL) ancestry-informative marker (AIM) panel. Human Mutation. 2010;31: 184–190. 10.1002/humu.21159 19953531

[13] Pena SDJ, Di Pietro G, Fuchshuber-Moraes M, Genro JP, Hutz MH, Kehdy F de SG, et al The Genomic Ancestry of Individuals from Different Geographical Regions of Brazil Is More Uniform Than Expected. Harpending H, editor. PLoS ONE. 2011;6: e17063 10.1371/journal.pone.0017063 21359226 PMC3040205

[14] Salzano FM, Sans M. Interethnic admixture and the evolution of Latin American populations. Genet Mol Biol. 2014;37: 151–170. 10.1590/s1415-47572014000200003 24764751 PMC3983580

[15] de Carvalho DC, Wanderley AV, dos Santos AMR, Fernandes MR, Cohen Lima de Castro A de N, Leitão LPC, et al Pharmacogenomics and variations in the risk of toxicity during the consolidation/maintenance phases of the treatment of pediatric B-cell leukemia patients from an admixed population in the Brazilian Amazon. Leukemia Research. 2018;74: 10–13. 10.1016/j.leukres.2018.09.003 30269037

[16] Sanchez E, Webb R, Rasmussen A, Kelly JA, Riba L, Kaufman KM, et al Genetically Determined Amerindian Ancestry Correlates with Increased Frequency of Risk Alleles for Systemic Lupus Erythematosus. Arthritis Rheum. 2010;62: 3722–3729. 10.1002/art.27753 20848568 PMC3078084

[17] Pinto P, Salgado C, Santos NPC, Santos S, Ribeiro-dos-Santos Â. Influence of Genetic Ancestry on INDEL Markers of NFKβ1, CASP8, PAR1, IL4 and CYP19A1 Genes in Leprosy Patients. PLOS Neglected Tropical Diseases. 2015;9: e0004050 10.1371/journal.pntd.0004050 26367014 PMC4569399

[18] Villar LM, Milagres FAP, Lampe E, Cruz HM, Scalioni L de P, Magalhães M de AFM, et al Determination of hepatitis B, C and D prevalence among urban and Amerindian populations from the Eastern Brazilian Amazon: a cross sectional study. BMC Infect Dis. 2018;18: 411 10.1186/s12879-018-3279-2 30126364 PMC6102873

[19] Ramos BR de A, D’Elia MPB, Amador MAT, Santos NPC, Santos SEB, da Cruz Castelli E, et al Neither self-reported ethnicity nor declared family origin are reliable indicators of genomic ancestry. Genetica. 2016;144: 259–265. 10.1007/s10709-016-9894-1 26984822

[20] Falush D, Stephens M, Pritchard JK. Inference of population structure using multilocus genotype data: dominant markers and null alleles. Mol Ecol Notes. 2007;7: 574–578. 10.1111/j.1471-8286.2007.01758.x 18784791 PMC1974779

[21] Falush D, Stephens M, Pritchard JK. Inference of population structure using multilocus genotype data: linked loci and correlated allele frequencies. Genetics. 2003;164: 1567–1587. 12930761 10.1093/genetics/164.4.1567 PMC1462648

[22] Palha T, Gusmão L, Ribeiro-Rodrigues E, Guerreiro JF, Ribeiro-dos-Santos Â, Santos S. Disclosing the Genetic Structure of Brazil through Analysis of Male Lineages with Highly Discriminating Haplotypes. PLOS ONE. 2012;7: e40007 10.1371/journal.pone.0040007 22808085 PMC3393733

[23] Moura RR de, Coelho AVC, Balbino V de Q, Crovella S, Brandão LAC. Meta-analysis of Brazilian genetic admixture and comparison with other Latin America countries. Am J Hum Biol. 2015;27: 674–680. 10.1002/ajhb.22714 25820814

[24] Glaziou P, Floyd K, Raviglione MC. Global Epidemiology of Tuberculosis. Semin Respir Crit Care Med. 2018;39: 271–285. 10.1055/s-0038-1651492 30071543

[25] Ministério da Saúde. Brasil Livre da Tuberculose: evolução dos cenários epidemiológicos e operacionais da doença. 2019. Available: https://portalarquivos2.saude.gov.br/images/pdf/2019/marco/22/2019-009.pdf

[26] Lindenau JD, Guimarães LSP, Friedrich DC, Hurtado AM, Hill KR, Salzano FM, et al Cytokine gene polymorphisms are associated with susceptibility to tuberculosis in an Amerindian population. Int J Tuberc Lung Dis. 2014;18: 952–957. 10.5588/ijtld.14.0060 25199010

[27] Longhi RMP, Zembrzuski VM, Basta PC, Croda J. Genetic polymorphism and immune response to tuberculosis in indigenous populations: a brief review. Braz J Infect Dis. 2013;17: 363–368. 10.1016/j.bjid.2012.11.001 23665009 PMC9427389

[28] Coimbra CEA, Basta PC. The burden of tuberculosis in indigenous peoples in Amazonia, Brazil. Trans R Soc Trop Med Hyg. 2007;101: 635–636. 10.1016/j.trstmh.2007.03.013 17467759

[29] Pereira F dos SCF, Guimarães RM, Lucidi AR, Brum DG, Paiva CLA, Alvarenga RMP. A systematic literature review on the European, African and Amerindian genetic ancestry components on Brazilian health outcomes. Sci Rep. 2019;9: 1–11. 10.1038/s41598-018-37186-2 31221977 PMC6586659

[30] Norris ET, Wang L, Conley AB, Rishishwar L, Mariño-Ramírez L, Valderrama-Aguirre A, et al Genetic ancestry, admixture and health determinants in Latin America. BMC Genomics. 2018;19 10.1186/s12864-017-4414-y 30537949 PMC6288849

[31] Chimusa ER, Zaitlen N, Daya M, Möller M, van Helden PD, Mulder NJ, et al Genome-wide association study of ancestry-specific TB risk in the South African Coloured population. Hum Mol Genet. 2014;23: 796–809. 10.1093/hmg/ddt462 24057671 PMC3888262

[32] Daya M, van der Merwe L, van Helden PD, Möller M, Hoal EG. The role of ancestry in TB susceptibility of an admixed South African population. Tuberculosis. 2014;94: 413–420. 10.1016/j.tube.2014.03.012 24832562

[33] Carvalho DC, Wanderley AV, Amador MAT, Fernandes MR, Cavalcante GC, Pantoja KBCC, et al Amerindian genetic ancestry and INDEL polymorphisms associated with susceptibility of childhood B-cell Leukemia in an admixed population from the Brazilian Amazon. Leukemia Research. 2015;39: 1239–1245. 10.1016/j.leukres.2015.08.008 26321572

[34] Lopes C de B, Barroso RFF, Burbano RMR, Garcia PA, Pinto PD do C, Santos NPCD, et al Effect of ancestry on interleukin-10 haplotypes in chronic periodontitis. Front Biosci (Elite Ed). 2017;9: 276–285. 10.2741/e802 28410151

[35] Augusto DG, Hollenbach JA, Petzl-Erler ML. A deep look at KIR-HLA in Amerindians: comprehensive meta-analysis reveals limited diversity of KIR haplotypes. Hum Immunol. 2015;76: 272–280. 10.1016/j.humimm.2015.01.025 25636566

[36] Bhatia KK, Black FL, Smith TA, Prasad ML, Koki GN. Class I HLA antigens in two long-separated populations: Melanesians and South Amerinds. Am J Phys Anthropol. 1995;97: 291–305. 10.1002/ajpa.1330970304 7573377

[37] Lindenau JD, Salzano FM, Guimarães LSP, Callegari-Jacques SM, Hurtado AM, Hill KR, et al Distribution patterns of variability for 18 immune system genes in Amerindians—relationship with history and epidemiology: Immune response SNPs in Amerindians. Tissue Antigens. 2013;82: 177–185. 10.1111/tan.12183 24032724

[38] Prugnolle F, Manica A, Balloux F. Geography predicts neutral genetic diversity of human populations. Curr Biol. 2005;15: R159–R160. 10.1016/j.cub.2005.02.038 15753023 PMC1800886

[39] Tsuneto LT, Probst CM, Hutz MH, Salzano FM, Rodriguez-Delfin LA, Zago MA, et al HLA class II diversity in seven Amerindian populations. Clues about the origins of the Aché. Tissue Antigens. 2003;62: 512–526. 10.1046/j.1399-0039.2003.00139.x 14617035

[40] Kidd P. Th1/Th2 balance: the hypothesis, its limitations, and implications for health and disease. Altern Med Rev. 2003;8: 223–246. 12946237

[41] Winek J, Demkow U, Rowińska-Zakrzewska E, Szołkowska M, Filewska M, Jagodziński J, et al [Comparison of Th1 and Th2 response in the blood of tuberculous patients and healthy contacts]. Pneumonol Alergol Pol. 2009;77: 446–452. 19890824

[42] Basta PC, de Sousa Viana PV. Determinants of tuberculosis in Indigenous people worldwide. Lancet Glob Health. 2019;7: e6–e7. 10.1016/S2214-109X(18)30525-4 30554763

[43] Garrido M da S, Bührer-Sékula S, Souza AB de, Ramasawmy R, Quincó P de L, Monte RL, et al Temporal distribution of tuberculosis in the State of Amazonas, Brazil. Rev Soc Bras Med Trop. 2015;48: 63–69. 10.1590/0037-8682-0055-2014 26061372

