# Supplementary material for: Amerindian genetic ancestry as a risk factor for tuberculosis in an amazonian population
Source: PLoS One. 2020 Jul 16;15(7):e0236033. doi: 10.1371/journal.pone.0236033 (PMC7365596; doi:10.1371/journal.pone.0236033)
Supplement: S1 Table — All statistical data were performed using the multivariate logistic regression model with the sex control variable. (DOCX) [file pone.0236033.s001.docx]

| **Ameridian** | | | | | |
| --- | --- | --- | --- | --- | --- |
| **Ancestry (%)** | **Case**  **(n)** | **Control**  **(n)** | **OR** | **IC (95%)** | ***p-value*** |
| 20-30% | 85 | 33 | 1.92 | 1.03 – 3.63 | 0.041 |
| 30-40% | 66 | 31 | 1.92 | 1.01 – 3.66 | 0.047 |
| 40-50% | 55 | 15 | 3.25 | 1.56 – 7.01 | 0.002 |
| 50-60% | 29 | 08 | 2.72 | 1.09 – 7.30 | 0.037 |
| 60-70% | 05 | 02 | 0.71 | 0.13 – 5.41 | 0.70 |
| 70-80% | 00 | 02 | 00 | NA – 2.16 | 0.981 |
