# Supplementary material for: Amerindian genetic ancestry as a risk factor for tuberculosis in an amazonian population
Source: PLoS One. 2020 Jul 16;15(7):e0236033. doi: 10.1371/journal.pone.0236033 (PMC7365596; doi:10.1371/journal.pone.0236033)
Supplement: S2 Table — (PDF) [file pone.0236033.s002.pdf]

SAMPLE	**GROUP**	**AGE**	**SEX**	**AFRICAN**	**EUROPEAN**	**AMERINDIAN**
TB01	1	42	0	0.570	0.227	0.203
TB02	1	71	1	0.387	0.351	0.261
TB03	1	55	1	0.157	0.393	0.450
TB04	1	63	1	0.174	0.611	0.215
TB05	1	39	1	0.340	0.519	0.141
TB06	1	39	0	0.413	0.317	0.270
TB07	1	7	1	0.279	0.475	0.245
TB08	1	NA	0	0.141	0.639	0.220
TB09	1	47	1	0.230	0.537	0.234
TB10	1	NA	1	0.176	0.614	0.210
TB11	1	66	0	0.126	0.575	0.299
TB12	1	42	1	0.164	0.331	0.505
TB13	1	55	0	0.249	0.388	0.362
TB14	1	85	1	0.636	0.147	0.217
TB15	1	84	1	0.156	0.515	0.329
TB16	1	64	0	0.327	0.397	0.276
TB17	1	37	0	0.168	0.270	0.561
TB18	1	NA	1	0.170	0.375	0.454
TB19	1	5	0	0.210	0.549	0.241
TB20	1	59	1	0.100	0.317	0.583
TB21	1	NA	1	0.147	0.566	0.287
TB22	1	35	0	0.353	0.272	0.374
TB23	1	37	0	0.315	0.525	0.160
TB24	1	36	0	0.314	0.361	0.325
TB25	1	NA	0	0.290	0.114	0.596
TB26	1	71	0	0.194	0.403	0.404
TB27	1	88	1	0.101	0.366	0.533
TB28	1	59	1	0.342	0.310	0.348
TB29	1	56	1	0.127	0.359	0.514
TB30	1	55	0	0.511	0.216	0.273
TB31	1	55	0	0.276	0.561	0.163
TB32	1	59	0	0.112	0.349	0.539
TB33	1	28	0	0.213	0.394	0.393
TB34	1	8	0	0.280	0.451	0.269
TB35	1	25	0	0.171	0.686	0.143
TB36	1	48	1	0.264	0.482	0.254
TB37	1	28	1	0.240	0.471	0.290
TB38	1	83	0	0.330	0.377	0.293
TB39	1	NA	0	0.331	0.343	0.325
TB40	1	61	1	0.182	0.435	0.383
TB41	1	63	0	0.177	0.375	0.448
TB42	1	36	1	0.184	0.571	0.244
TB43	1	44	1	0.236	0.478	0.286
TB44	1	6	1	0.199	0.299	0.503
TB45	1	7	0	0.323	0.437	0.241
TB46	1	38	1	0.152	0.396	0.451
TB47	1	62	0	0.115	0.295	0.590

TB48	1	72	1	0.131	0.400	0.469
TB49	1	63	1	0.190	0.450	0.361
TB50	1	77	0	0.266	0.468	0.265
TB51	1	3	1	0.231	0.459	0.310
TB52	1	55	1	0.317	0.276	0.407
TB53	1	38	1	0.071	0.267	0.662
TB54	1	48	1	0.195	0.550	0.255
TB55	1	64	0	0.252	0.260	0.488
TB56	1	28	1	0.168	0.541	0.291
TB57	1	45	0	0.257	0.580	0.163
TB58	1	65	1	0.437	0.325	0.238
TB59	1	21	0	0.193	0.590	0.217
TB60	1	47	1	0.214	0.491	0.295
TB61	1	57	1	0.122	0.509	0.369
TB62	1	54	0	0.139	0.695	0.166
TB63	1	88	1	0.376	0.362	0.262
TB64	1	52	1	0.371	0.395	0.234
TB65	1	61	1	0.256	0.194	0.550
TB66	1	31	0	0.220	0.538	0.243
TB67	1	57	0	0.645	0.162	0.192
TB68	1	33	1	0.252	0.476	0.272
TB69	1	NA	1	0.364	0.433	0.203
TB70	1	NA	1	0.340	0.454	0.205
TB71	1	68	0	0.125	0.520	0.354
TB72	1	29	0	0.299	0.529	0.173
TB73	1	45	1	0.220	0.544	0.237
TB74	1	44	1	0.381	0.254	0.365
TB75	1	48	0	0.328	0.276	0.396
TB76	1	42	1	0.307	0.488	0.204
TB77	1	52	0	0.185	0.334	0.481
TB78	1	53	1	0.672	0.137	0.191
TB79	1	31	1	0.285	0.523	0.192
TB80	1	23	1	0.127	0.414	0.459
TB81	1	45	0	0.156	0.443	0.401
TB82	1	49	0	0.270	0.527	0.203
TB83	1	24	0	0.168	0.416	0.416
TB84	1	67	0	0.307	0.425	0.268
TB85	1	5	1	0.108	0.781	0.111
TB86	1	62	1	0.150	0.645	0.204
TB87	1	38	1	0.343	0.260	0.397
TB88	1	51	1	0.102	0.530	0.368
TB89	1	44	0	0.244	0.394	0.362
TB90	1	49	0	0.210	0.586	0.204
TB91	1	7	0	0.249	0.316	0.434
TB92	1	35	1	0.194	0.436	0.370
TB93	1	27	1	0.410	0.448	0.142
TB94	1	NA	0	0.249	0.315	0.436
TB95	1	47	0	0.217	0.324	0.459

TB96	1	34	1	0.573	0.318	0.109
TB97	1	46	1	0.143	0.465	0.392
TB98	1	48	1	0.414	0.162	0.425
TB99	1	51	1	0.350	0.447	0.203
TB100	1	72	0	0.186	0.228	0.587
TB101	1	32	0	0.133	0.562	0.305
TB102	1	5	1	0.300	0.435	0.265
TB103	1	45	0	0.094	0.358	0.548
TB104	1	5	0	0.267	0.574	0.159
TB105	1	NA	0	0.230	0.207	0.563
TB106	1	65	0	0.162	0.383	0.455
TB107	1	46	0	0.166	0.327	0.507
TB108	1	4	1	0.097	0.581	0.322
TB109	1	56	1	0.143	0.444	0.413
TB110	1	32	0	0.209	0.388	0.402
TB111	1	51	1	0.170	0.440	0.390
TB112	1	84	0	0.115	0.762	0.123
TB113	1	26	0	0.153	0.463	0.383
TB114	1	66	0	0.126	0.303	0.570
TB115	1	25	1	0.180	0.222	0.597
TB116	1	49	0	0.107	0.554	0.338
TB117	1	77	0	0.221	0.388	0.391
TB118	1	54	0	0.221	0.517	0.262
TB119	1	55	0	0.228	0.349	0.423
TB120	1	59	0	0.102	0.486	0.412
TB121	1	34	1	0.285	0.207	0.508
TB122	1	5	0	0.378	0.468	0.153
TB123	1	NA	1	0.241	0.149	0.610
TB124	1	39	1	0.428	0.275	0.297
TB125	1	85	1	0.302	0.454	0.244
TB126	1	57	1	0.090	0.503	0.407
TB127	1	NA	1	0.364	0.290	0.345
TB128	1	19	1	0.551	0.282	0.167
TB129	1	43	0	0.228	0.438	0.333
TB130	1	53	0	0.186	0.344	0.470
TB131	1	79	1	0.260	0.411	0.328
TB132	1	36	0	0.231	0.462	0.307
TB133	1	38	0	0.334	0.465	0.200
TB134	1	6	0	0.249	0.556	0.195
TB135	1	27	1	0.124	0.653	0.223
TB136	1	51	1	0.347	0.416	0.237
TB137	1	NA	0	0.135	0.303	0.561
TB138	1	47	0	0.16	0.447	0.393
TB139	1	26	0	0.168	0.424	0.408
TB140	1	NA	0	0.16	0.487	0.354
TB141	1	NA	0	0.195	0.519	0.286
TB142	1	35	1	0.579	0.174	0.247
TB143	1	27	0	0.19	0.622	0.188

TB144	1	35	0	0.178	0.604	0.217
TB145	1	29	1	0.103	0.522	0.375
TB146	1	43	1	0.116	0.409	0.475
TB147	1	NA	0	0.258	0.605	0.137
TB148	1	NA	0	0.422	0.279	0.299
TB149	1	73	1	0.241	0.495	0.264
TB150	1	NA	0	0.227	0.529	0.245
TB151	1	7	1	0.392	0.17	0.438
TB152	1	59	1	0.237	0.637	0.126
TB153	1	72	1	0.11	0.55	0.34
TB154	1	NA	1	0.163	0.618	0.219
TB155	1	54	0	0.353	0.435	0.212
TB156	1	69	1	0.174	0.598	0.228
TB157	1	77	0	0.188	0.547	0.266
TB158	1	NA	0	0.162	0.537	0.301
TB159	1	66	0	0.449	0.294	0.257
TB160	1	45	0	0.395	0.237	0.367
TB161	1	66	1	0.455	0.264	0.282
TB162	1	NA	0	0.318	0.521	0.161
TB163	1	NA	0	0.141	0.386	0.473
TB164	1	25	1	0.183	0.297	0.52
TB165	1	46	0	0.224	0.291	0.485
TB166	1	NA	0	0.322	0.323	0.355
TB167	1	NA	0	0.308	0.513	0.179
TB168	1	NA	0	0.182	0.601	0.217
TB169	1	NA	0	0.235	0.374	0.392
TB170	1	64	0	0.192	0.367	0.441
TB171	1	27	0	0.22	0.336	0.444
TB172	1	74	0	0.218	0.611	0.171
TB173	1	38	0	0.202	0.355	0.443
TB174	1	51	1	0.299	0.302	0.399
TB175	1	NA	0	0.205	0.465	0.329
TB176	1	51	0	0.227	0.392	0.381
TB177	1	35	0	0.299	0.387	0.314
TB178	1	NA	0	0.3	0.312	0.389
TB179	1	NA	0	0.193	0.637	0.169
TB180	1	6	0	0.334	0.438	0.228
TB181	1	51	0	0.512	0.21	0.278
TB182	1	76	1	0.276	0.385	0.338
TB183	1	53	1	0.357	0.29	0.353
TB184	1	66	0	0.218	0.629	0.154
TB185	1	NA	1	0.444	0.305	0.251
TB186	1	NA	0	0.415	0.256	0.329
TB187	1	48	1	0.159	0.362	0.48
TB188	1	NA	0	0.155	0.397	0.448
TB189	1	29	0	0.208	0.438	0.354
TB190	1	NA	0	0.193	0.288	0.519
TB191	1	NA	1	0.119	0.478	0.402

TB192	1	NA	1	0.257	0.261	0.481
TB193	1	4	1	0.318	0.47	0.212
TB194	1	53	1	0.288	0.452	0.26
TB195	1	NA	0	0.161	0.43	0.409
TB196	1	6	1	0.149	0.379	0.472
TB197	1	NA	0	0.290	0.266	0.444
TB198	1	NA	1	0.101	0.294	0.605
TB199	1	5	1	0.139	0.309	0.552
TB200	1	43	1	0.235	0.342	0.423
TB201	1	69	1	0.259	0.369	0.371
TB202	1	52	1	0.356	0.406	0.238
TB203	1	NA	0	0.305	0.423	0.273
TB204	1	45	0	0.289	0.512	0.199
TB205	1	57	0	0.153	0.338	0.509
TB206	1	27	0	0.219	0.396	0.386
TB207	1	61	1	0.618	0.226	0.156
TB208	1	NA	0	0.148	0.598	0.253
TB209	1	55	0	0.405	0.379	0.216
TB210	1	NA	1	0.332	0.168	0.500
TB211	1	89	0	0.204	0.422	0.375
TB212	1	NA	0	0.131	0.368	0.501
TB213	1	77	1	0.385	0.149	0.466
TB214	1	NA	0	0.592	0.220	0.187
TB215	1	NA	1	0.451	0.354	0.195
TB216	1	NA	0	0.451	0.404	0.145
TB217	1	NA	1	0.454	0.380	0.166
TB218	1	NA	0	0.129	0.547	0.324
TB219	1	54	1	0.335	0.336	0.329
TB220	1	NA	0	0.402	0.312	0.287
TB221	1	82	1	0.214	0.518	0.268
TB222	1	NA	1	0.137	0.332	0.531
TB223	1	NA	1	0.091	0.301	0.608
TB224	1	72	0	0.164	0.350	0.486
TB225	1	NA	0	0.176	0.342	0.482
TB226	1	NA	1	0.284	0.418	0.299
TB227	1	NA	0	0.207	0.227	0.567
TB228	1	NA	1	0.156	0.382	0.462
TB229	1	NA	0	0.371	0.332	0.297
TB230	1	3	0	0.406	0.284	0.310
TB231	1	67	0	0.372	0.437	0.191
TB232	1	NA	0	0.426	0.231	0.343
TB233	1	53	0	0.175	0.597	0.228
TB234	1	28	0	0.121	0.394	0.485
TB235	1	58	0	0.248	0.400	0.352
TB236	1	NA	0	0.356	0.264	0.380
TB237	1	67	1	0.175	0.609	0.216
TB238	1	NA	1	0.133	0.455	0.412
TB239	1	NA	0	0.144	0.408	0.448

TB240	1	36	0	0.134	0.332	0.534
TB241	1	NA	1	0.152	0.322	0.526
TB242	1	NA	1	0.169	0.526	0.305
TB243	1	NA	0	0.055	0.511	0.435
TB244	1	33	1	0.211	0.139	0.649
TB245	1	NA	1	0.093	0.620	0.286
TB246	1	NA	1	0.246	0.444	0.310
TB247	1	NA	1	0.274	0.347	0.379
TB248	1	NA	0	0.203	0.481	0.316
TB249	1	26	1	0.347	0.342	0.310
TB250	1	25	0	0.264	0.355	0.381
TB251	1	36	1	0.300	0.432	0.267
TB252	1	58	1	0.130	0.733	0.138
TB253	1	22	0	0.330	0.515	0.155
TB254	1	54	1	0.247	0.546	0.207
TB255	1	75	0	0.649	0.200	0.151
TB256	1	34	0	0.397	0.352	0.251
TB257	1	23	0	0.305	0.323	0.372
TB258	1	17	1	0.167	0.415	0.418
TB259	1	2	1	0.294	0.332	0.374
TB260	1	55	0	0.226	0.535	0.239
TB261	1	69	1	0.262	0.313	0.425
TB262	1	28	0	0.190	0.438	0.372
TB263	1	37	1	0.175	0.406	0.419
TB264	1	21	1	0.193	0.481	0.326
TB265	1	4	1	0.148	0.320	0.533
TB266	1	NA	1	0.203	0.628	0.168
TB267	1	45	1	0.410	0.389	0.201
TB268	1	NA	1	0.200	0.551	0.249
TB269	1	NA	0	0.183	0.376	0.441
TB270	1	NA	1	0.385	0.460	0.156
TB271	1	25	0	0.159	0.391	0.450
TB272	1	64	0	0.219	0.500	0.281
TB273	1	NA	1	0.213	0.311	0.477
TB274	1	NA	1	0.550	0.222	0.227
TB275	1	NA	1	0.303	0.280	0.417
TB276	1	NA	1	0.229	0.226	0.544
TB277	1	NA	0	0.284	0.560	0.157
TB278	1	NA	0	0.318	0.480	0.202
TB279	1	NA	1	0.433	0.274	0.293
TB280	1	NA	1	0.409	0.213	0.378
CONT01	0	65	0	0.205	0.221	0.574
CONT02	0	46	0	0.171	0.583	0.246
CONT03	0	45	0	0.092	0.5	0.408
CONT04	0	5	0	0.135	0.672	0.193
CONT05	0	39	0	0.233	0.346	0.421
CONT06	0	52	0	0.114	0.735	0.151
CONT07	0	69	0	0.176	0.567	0.257

CONT08	0	46	0	0.203	0.64	0.157
CONT09	0	45	0	0.402	0.412	0.186
CONT10	0	53	0	0.227	0.486	0.288
CONT11	0	69	0	0.072	0.55	0.378
CONT12	0	62	1	0.131	0.569	0.3
CONT13	0	53	0	0.245	0.518	0.237
CONT14	0	64	0	0.069	0.587	0.344
CONT15	0	57	0	0.178	0.43	0.392
CONT16	0	56	0	0.392	0.438	0.17
CONT17	0	48	0	0.226	0.665	0.109
CONT18	0	53	0	0.215	0.574	0.211
CONT19	0	69	1	0.155	0.688	0.156
CONT20	0	49	0	0.402	0.435	0.162
CONT21	0	36	0	0.09	0.758	0.151
CONT22	0	52	1	0.148	0.532	0.32
CONT23	0	55	0	0.113	0.356	0.531
CONT24	0	47	1	0.168	0.316	0.516
CONT25	0	45	0	0.185	0.662	0.153
CONT26	0	67	0	0.398	0.487	0.116
CONT27	0	56	0	0.136	0.526	0.338
CONT28	0	58	0	0.161	0.468	0.37
CONT29	0	5	0	0.123	0.582	0.295
CONT30	0	61	0	0.32	0.474	0.207
CONT31	0	44	0	0.137	0.373	0.489
CONT32	0	57	0	0.185	0.322	0.493
CONT33	0	52	0	0.303	0.271	0.426
CONT34	0	66	0	0.278	0.162	0.56
CONT35	0	5	0	0.142	0.407	0.45
CONT36	0	48	0	0.288	0.532	0.179
CONT37	0	47	0	0.307	0.386	0.307
CONT38	0	57	0	0.146	0.402	0.452
CONT39	0	54	0	0.126	0.477	0.397
CONT40	0	63	0	0.641	0.218	0.141
CONT41	0	59	0	0.196	0.493	0.312
CONT42	0	49	1	0.144	0.641	0.215
CONT43	0	47	0	0.23	0.583	0.187
CONT44	0	58	0	0.279	0.344	0.377
CONT45	0	51	0	0.129	0.508	0.364
CONT46	0	61	0	0.057	0.874	0.07
CONT47	0	55	0	0.064	0.773	0.164
CONT48	0	54	0	0.112	0.812	0.076
CONT49	0	56	0	0.153	0.507	0.34
CONT50	0	47	0	0.139	0.527	0.334
CONT51	0	6	0	0.164	0.517	0.319
CONT52	0	53	0	0.248	0.57	0.182
CONT53	0	47	0	0.49	0.398	0.112
CONT54	0	61	0	0.305	0.473	0.222
CONT55	0	6	0	0.218	0.5	0.282

CONT56	0	56	1	0.608	0.219	0.173
CONT57	0	49	0	0.134	0.525	0.341
CONT58	0	4	0	0.131	0.404	0.465
CONT59	0	45	0	0.286	0.514	0.2
CONT60	0	62	1	0.162	0.569	0.269
CONT61	0	49	0	0.116	0.782	0.103
CONT62	0	38	0	0.267	0.522	0.21
CONT63	0	37	0	0.397	0.447	0.156
CONT64	0	59	0	0.362	0.171	0.466
CONT65	0	53	0	0.238	0.222	0.54
CONT66	0	51	0	0.52	0.195	0.285
CONT67	0	62	0	0.181	0.428	0.39
CONT68	0	53	0	0.239	0.439	0.323
CONT69	0	53	1	0.146	0.24	0.615
CONT70	0	35	1	0.208	0.654	0.137
CONT71	0	43	0	0.251	0.428	0.321
CONT72	0	44	0	0.142	0.768	0.09
CONT73	0	48	0	0.164	0.49	0.346
CONT74	0	NA	0	0.226	0.276	0.498
CONT75	0	NA	0	0.208	0.482	0.311
CONT76	0	62	0	0.348	0.353	0.299
CONT77	0	68	0	0.725	0.13	0.145
CONT78	0	46	0	0.166	0.753	0.08
CONT79	0	35	0	0.295	0.353	0.352
CONT80	0	61	0	0.101	0.701	0.198
CONT81	0	48	0	0.115	0.572	0.313
CONT82	0	46	0	0.13	0.591	0.279
CONT83	0	32	0	0.316	0.452	0.233
CONT84	0	58	0	0.206	0.483	0.31
CONT85	0	51	0	0.136	0.778	0.086
CONT86	0	45	0	0.308	0.543	0.15
CONT87	0	65	1	0.11	0.629	0.26
CONT88	0	44	0	0.24	0.388	0.371
CONT89	0	71	1	0.111	0.742	0.148
CONT90	0	47	1	0.236	0.433	0.332
CONT91	0	57	0	0.214	0.531	0.255
CONT92	0	56	0	0.283	0.537	0.18
CONT93	0	36	0	0.252	0.376	0.371
CONT94	0	52	0	0.165	0.571	0.264
CONT95	0	6	0	0.128	0.755	0.117
CONT96	0	52	0	0.128	0.754	0.118
CONT97	0	56	0	0.24	0.622	0.138
CONT98	0	6	0	0.105	0.775	0.121
CONT99	0	43	0	0.291	0.536	0.173
CONT100	0	74	0	0.242	0.69	0.068
CONT101	0	47	0	0.126	0.41	0.464
CONT102	0	53	0	0.338	0.331	0.331
CONT103	0	54	0	0.207	0.728	0.065

CONT104	0	34	0	0.212	0.643	0.145
CONT105	0	48	0	0.088	0.634	0.278
CONT106	0	63	0	0.342	0.187	0.472
CONT107	0	63	0	0.287	0.615	0.098
CONT108	0	46	0	0.18	0.412	0.407
CONT109	0	45	0	0.354	0.54	0.106
CONT110	0	62	0	0.205	0.589	0.206
CONT111	0	52	0	0.139	0.57	0.291
CONT112	0	38	1	0.286	0.353	0.362
CONT113	0	38	0	0.238	0.422	0.34
CONT114	0	34	0	0.319	0.459	0.222
CONT115	0	56	0	0.185	0.582	0.232
CONT116	0	57	0	0.182	0.474	0.344
CONT117	0	54	0	0.205	0.558	0.237
CONT118	0	57	0	0.088	0.597	0.316
CONT119	0	63	0	0.249	0.56	0.191
CONT120	0	45	0	0.312	0.501	0.188
CONT121	0	48	1	0.309	0.169	0.521
CONT122	0	34	0	0.578	0.189	0.233
CONT123	0	34	0	0.184	0.583	0.233
CONT124	0	62	1	0.135	0.313	0.552
CONT125	0	59	0	0.098	0.778	0.124
CONT126	0	46	1	0.19	0.561	0.249
CONT127	0	NA	0	0.133	0.1	0.767
CONT128	0	NA	1	0.243	0.133	0.624
CONT129	0	NA	0	0.107	0.455	0.438
CONT130	0	NA	0	0.154	0.14	0.707
CONT131	0	54	0	0.221	0.553	0.226
CONT132	0	65	0	0.134	0.762	0.104
CONT133	0	NA	1	0.142	0.442	0.416
CONT134	0	32	1	0.18	0.618	0.202
CONT135	0	58	0	0.112	0.676	0.212
CONT136	0	NA	1	0.08	0.36	0.559
CONT137	0	NA	1	0.172	0.54	0.287
CONT138	0	47	0	0.137	0.711	0.152

NA- No annotation
